# Resolutions of Respect to Dr. George H. Cushing, M.D., D.D.S.

**Published:** 1900-07

**Authors:** 


### RESOLUTIONS OF RESPECT TO DR. GEORGE H. CUSHING, M.D., D.D.S.

It has come to the notice of the Chicago Dental Society that our beloved brother and fellow-practitioner, Dr. George H. Cushing, one of the founders of this Society, departed this life May 25, 1900, at Los Angeles, California; therefore be it

*Resolved,* That we mourn his loss as personal, because every member of the Society knew him and loved him as a friend, counsellor, and guide. This is no ordinary expression from a committee appointed to draw a memorial, but one where every member feels that it is a personal loss to no longer see and feel the presence of one so much beloved; be it further

*Resolved,* That in Dr. Cushing we mourn the loss of one who was in every sense an inspiration to do one's best in all the complex hours of life's duties. He was monitor, teacher, and helper in all emergencies. His life was devoted to his profession in a sense little understood by the thoughtless, and his influence for good was far-reaching and always to be relied upon.

We feel that in this feeble tribute to the vast labors he performed for forty years we fall short in our estimation of them because no one was more stanch in his devotion to private or public duties than our departed friend. He had his faults, but they were so obscured by his noble and gentle nature that few knew them; so they must be in an oblivion so perfect that only his most bitter enemy may recall them. For us he was the pattern of the kindly professional gentleman and devoted friend.

We extend to his family our condolence and the expression of our sorrow. We will all go to his last resting-place feeling that life is only a brief day and that we will meet him again in the near future, where all is peace and serenity.

*Resolved,* That a copy of this tribute be sent to the family of the deceased and to the dental journals for publication.

A. W. Harlan.
Truman W. Brophy.
J. W. Wassall.

